# PubMed indexation for the European Heart Journal – Digital Health: a small step for the European Heart Journal family, a giant leap in the digital future of cardiovascular disease management

**DOI:** 10.1093/ehjdh/ztad013

**Published:** 2023-02-24

**Authors:** Peter de Jaegere, Robert van der Boon, Joost Lumens, Nico Bruining

**Affiliations:** Department of Cardiology, Erasmus MC, Dr Molewaterplein 40, 3015 GD Rotterdam, The Netherlands; Department of Cardiology, Erasmus MC, Dr Molewaterplein 40, 3015 GD Rotterdam, The Netherlands; CARIM School for Cardiovascular Diseases, Maastricht University Medical Center, Maastricht, The Netherlands; Department of Cardiology, Erasmus MC, Dr Molewaterplein 40, 3015 GD Rotterdam, The Netherlands

In June 2020, the European Society of Cardiology (ESC) appointed Dr Nico Bruining (computer scientist), heading the Department of Clinical and Experimental Information Processing at the Department of Cardiology of the Erasmus MC, the editorship of the *European Heart Journal – Digital Health* (EHJ-DH). As such, the Department of Cardiology of the Erasmus MC, Rotterdam, the Netherlands, became de facto the editorial office. Undersigned (interventional cardiologist) became deputy editor. We were quickly accompanied by Prof. Dr Joost Lumens (biomedical engineer) from the CARIM School for Cardiovascular Diseases, Maastricht University Medical Center, and more recently by a cardiologist with keen interest in innovation in the domain of heart failure, DR Robert van der Boon. This foursome has the honour and task to respond to the mission of the ESC, namely, the reduction of the burden of cardiovascular disease via the fostering of Digital Health, thereby, enhancing prevention and rendering diagnosis/treatment of cardiovascular disease more personalized and precise as well as timelier and, henceforth, more efficient.^1,2^

Digital transformation of health & care is the logic consequence of a world in which an object or analogue signal whatever its source, physical and/or chemical properties are converted into a numerical format using a binary code (i.e. 0 and 1) that is readable by a computer and easy to transfer at an astonishing speed without degradation. Binary digits or bits are the smallest increment of data on a computer that are assembled into groups of eight to form a byte.^3–5^

The amount of (biomedical) information is astounding and still largely undetected whilst already overwhelming us due to the incessant growth in novel sophisticated (biomedical) capturing or detection devices from implantable devices to tattoos, plus the development of technological innovations for treatment planning, execution, and monitoring (wired or wireless).^6–10^

‘Standard’ language or the algorithm with which we transfer information does not suffice to describe its amount. For instance and from a bigger perspective, it is estimated that by 2030, the world will generate approximately a yottabyte of data per year or 10^24^ bytes.^11^ Yotta already fails to define the incessant increase in data. Prefixes such as ronna and quetta (10^27^ and 10^30^) are needed with ronto and quecto to denote the opposite (10^−27^ and 10^−30^). To illustrate, we have the following mind-boggling exponential qualities of the physical world (data source): the Earth weighs around one ronnagram and an electron’s mass is about one quectogram.^11^

The prodigious generation and, hence, an ocean of multidimensional data demand novel data processing and integration techniques for the extraction of the features of signals relevant for the objective and devoid of ‘noise’, their transfer, and stockage, followed by analysis to display or representation. Current computer systems may not be up to the task any longer. As a historical example, in the 1960’s, the Saturn V computer operated at a rate of 512 kilobits per second.^12^ It is clear that we need more space–time–analysis capacity/power to fulfil the DH transformation in cardiovascular medicine.^13^ Additionally, our binary world and way of thinking may prove to have become obsolete.

For that matter, we may return for inspiration back to our brain that already has been the spur of computer design and artificial intelligence, in particular deep learning. At variance with a digital computer, but similar to the human brain, quantum computers can search in disconnected data sets and answer unasked questions in an unmatched time span.^14–16^ If true and if quantum computing will prove to fulfil our hopes and expectations, the process of the DH chain and use of its outcome will unavoidably request human supervision from both an intellectual and ethical perspective.^5,17^ After all, it is hypothesized that the human brain contains 100 billion q-bits and, hence, more powerful than all the digital computers in the world combined.^18^ The late Steven Weinberg, a Nobel Prize winner in Physics 1979, may be right when he said ‘we do not seem to be becoming to the end of our intellectual resources’.^19^ This implies that the humankind is, in principle, capable of solving every problem provided that we have the will to do so. Will and intelligence, however, will not avail in the lack or absence of the insight or emotional intelligence that the above mandates a ‘civilized’ conduct; a conduct based upon the moral values that have been constructed during centuries of (self) reflection and, thus, the development of a world in which freedom of thinking and acting accordingly via speech, writing, research, art, … is its cornerstone. Such a conduct is to be guided by the individual’s ‘logos’ or internal moral compass (constitution) and controlled and protected at a society level by its constitution and independent institutions.

The editorial board of this journal sees it as its responsibility to reach out and connect an indiscriminate array of professionals of singular backgrounds and invite them to submit their work or thoughts via the various formats of the article types this journal offers. Via publishing and exchange, we seek transfer of knowledge plus discussion and hope that this journal may serve as flagship. The open-access nature of the EHJ-DH is a logical choice. We know that one may see this differently and that some evenly openly oppose. In response, we kindly remind that open access is in line with the bidding of society besides the fact that it is the best way to bring information to all and stimulate discussion whilst acknowledging that economic barriers need to be addressed in various geographic areas.

Given a newborn in a plethora of medical journals, we reached PubMed indexation in a time span of approximately two years after the first issue that was published online in November 2020. We express our sincere appreciation and gratitude to all clinicians, scientists, and other healthcare professionals who support the journal by submitting their work or by sharing their thoughts, insights, … via article types. Likewise, we express great respect and again a sincere ‘thank you’ to all reviewers who dedicate their time, expertise, and commitment to improve the work submitted, and, consequently, the quality of this journal. One should not forget that a review process is most often not only a single but a repeated effort. Reviewers are consulted again to verify the responses to their comments and eventually for (an)other critical revision(s) until a final decision can be made by the editorial board. Last but not least, this journal would not have been possible without the outstanding support of the publisher’s team (Oxford University Press).

Science Citation Index will be the next landmark. Yet, this editorial board and future members will—above all—commit itself to acquire and publish original research and review papers next to teaching sessions and other formats of papers that are relevant to all stakeholders in cardiovascular medicine, thereby, meeting the task given by the ESC to us that is concisely captured by its mission statement formulated above. Science is one of the numerous methods or tools for the creation of a better world, a world in which every living has and/or receives the opportunity to blossom and flourish in freedom, safety, and prosperity of the body, mind, and soul in respect for its non-living environment, both prevented and protected from disaster and/or destruction.^20^ As 2023 comes to a close and as we have to face the alarming and incomprehensible views and actions by some, it is good to remember Dr M. L. King and his call for freedom to be rang from every state, city, hamlet, … so that we may become united irrespective of our beliefs, backgrounds, … It is in freedom that human creativity best flourishes and the best innovations will emerge. Freedom and truth are invariably connected to one another. If one cannot think, speak, or write in freedom, one cannot proclaim the truth.^21–24^
